# Learners’ experiences of an enhanced surgical skills training program for family physicians

**Published:** 2018-11-12

**Authors:** Jude Kornelsen, Brian Geller, Fred Janke, Stuart Iglesias

**Affiliations:** 1 Centre for Rural Health Research, Department of Family Practice, University of British Columbia, British Columbia, Canada; 2 Department of Family Medicine, University of Saskatchewan, Canada; 3 Office of Rural and Regional Health, Division of Community Engagement, Faculty of Medicine and Dentistry, University of Alberta, Alberta, Canada; 4 Rural Coordination Centre of BC, British Columbia, Canada

## Abstract

**Background:**

Family Physicians with Enhanced Surgical Skills (FPESS) have sustained rural operative care, including local access to caesarean section, in many communities across rural Canada and internationally. The contemporary role of FPESS within the health system, however, has not been without challenges. The 12-month Prince Albert Enhanced Surgical Skills (ESS) program intakes two learners a year and is one of only two accredited programs in Canada offering a scope of surgical practice beyond operative delivery.

**Methods:**

This paper highlights the results of an evaluation of graduates’ experiences of training and the post-training environment. Graduates were practicing in Western and Northern Canada after completing the ESS training program, specifically in British Columbia, Alberta, Manitoba, and the Northwest Territories.

**Results:**

Findings suggest the overall success of the program in meeting learners’ needs. There was a close match between the training curriculum and post-training practice.

**Conclusion:**

The findings from the post training experience suggest that sustainability of ESS is linked to 1) creating pathways to privileges between the ESS community and the Health Authorities, 2) building functional and trusting relationships with surgical specialists, and 3) creating a web of accessible effective rurally appropriate surgical Continuing Professional Development (CDP). Ongoing CPD is identified as essential in increasing the comfort of FPESS.

## Introduction

Both Canadian and International scholars have noted the diversity of conditions cared for within a low volume generalist model .^1-5^ Evidence suggests the need for practitioners with a broad range of competencies, including caesarean section, to meet the surgical needs of rural communities.^6-8^ In most settings, the low frequency of procedural care makes it an unlikely place for specialist practice given the movement away from “solo” practice and towards group practices with sustainable on-call rotations.^9^ The same challenges of low procedural volume have an impact on Family Physicians with Enhanced Surgical Skills (FPESS) practice, particularly when training has been recent. This is often addressed through rotating practice in high-volume regional or tertiary centres or through overseas work and addressed locally through on-going interprofessional Continuing Professional Development (CPD). Recent innovations in British Columbia have led to the introduction of real presence technology, which enables virtual linkages between rural surgical services with regional referral sites for virtual procedural learning and urgent consult if necessary. These strategies may mitigate the practice challenges in low volume centres; however, careful attention to local process for Continuous Quality Improvement and process evaluation are essential for system accountability.

Where specialist practice exists, it is often supported by a family physician with Enhanced Surgical Skills (FPESS) to reduce the burden of continuous on call. In rural communities in Western and Northern Canada, many of the surgical needs are met by FPESS in partnership with general practitioner (GP) anesthetists. This model of generalism plays well in settings that may not have the volume to support a full-time proceduralist but still have the need for some local care.

Support for Enhanced Surgical Skills for Canada’s rural family physicians is driven by the evidence based policy framework to sustain rural maternity care programs close to home. The recent recognition of the critical role played by robust local surgical programs in rural maternity care, as well as the importance of local surgical first responders, has been endorsed in the Joint Position Paper on Rural Surgery and Operative Delivery.^10^

The contemporary role of FPESS within the health system, however, has not been without challenges due to the lack of 1) national training standards supported by Canada’s two licensing Colleges, 2) on-going continuing medical education, and 3) supportive interprofessional relationships with specialist colleagues.^11^ These challenges are well-known among the Enhanced Surgical Skills (ESS) community. Understanding program participants’ experiences during training and post-program can lead to responsive adjustments to the program and awareness of ways pentagram partners (policy and decision-makers, health care administrators, care providers, communities, and researchers) can increase their support of rural surgical care.

The University of Saskatchewan (U of S) convened an Enhanced Surgical Skills Retreat in Prince Albert in September 2017 with the purpose of examining the first 10 years of its ESS program. Attending were graduates, faculty, researchers, postgraduate education, and representatives from specialty societies, Canada’s two licensing Colleges, and Health Authorities. The ESS program invited the University of British Columbia Centre for Rural Health Research to interview the graduates on their training and post-training experience

The history of training for rural FPESS identifies two pathways: 1) informal, in-person mentorship, most often as a one off effort that consists of only one interaction; and 2) a formal 12-month postgraduate accredited program. The University of Alberta had a 12-month training program based in Grande Prairie between 1990 and 2000. From 2007 to 2017, the University of Saskatchewan in Prince Albert was the only Canadian ESS program offering surgical training across a broad scope of obstetrical and general surgery procedures. Specialists worked to develop the skills of ESS residents through a curriculum of practical experience in the operating room (OR). The program was structured around six months of general surgical training and six months of obstetrical and gynecological training. Residents often spent time in other specialty areas (e.g., orthopaedics) as per individual and community interests and needs. There are three other 3-6 month ESS programs in Canada that offer a skill set restricted to operative delivery. A full scope ESS program has recently been started in Fort McMurray, Alberta through the University of Alberta.

Although there is a growing body of research evidence on the importance of a rural surgical workforce,^12^ rural-specific competencies^13,14^ and education-based mechanisms for meeting rural surgical need,^15^ most of the literature focuses on the specialist surgical workforce. There is scant research on the training of FPESS, and no program evaluations of the efficacy of training curricula. The research that does exist focuses on experiences of less formal training^11^ and experiences of practice.^7^ The former found significant issues around the lack of collegial and health authority support for the practice, the lack of recognized and respected credentials, the lack of formal training avenues, and the lack of effective accessible CPD.

A 2015 Canadian consensus effort proposed a curriculum leading to core competencies selected from “historical skill sets in which ESS physicians have provided services, the skill sets for which there is good research evidence on the outcomes and safety of appropriately trained ESS physicians performing these procedures… and the present University of Saskatchewan R3 ESS training program.”^16^ The authors suggested that the call for a core curriculum of defined competencies will lead to a generic and portable skill set which will be one step towards the stability of FPESS. It is encouraging that at the time of writing the College of Family Physicians of Canada (CFPC) and the Royal College of Physicians and Surgeons of Canada (RCPSC) were collaborating to translate the curriculum into the core competencies and national training standards for ESS.

Research from Australia examined the effect of a surgical skills course on confidence levels of rural general practitioners^17^ and found short-course training improved confidence levels for practitioners who had already completed training. The authors noted the difficulty for rural GPs who want to increase or maintain their surgical skills, namely the difficulty in leaving the community due to scarce locum coverage. Despite the availability of surgical training in Australia, however, and the qualifications offered by the Royal Australian College of General Practitioners (RACGP) and the Australian College of Rural and Remote Medicine (ACRRM), the training programs are not recognized by the Royal Australian College of Surgeons. The authors of a 2014 study note that due to this situation, the qualifications “provide little or no benefit in granting surgical privileges in rural hospitals.”^18^ Concomitantly, the authors suggested that any training pathway for this group “needs to be developed in collaboration with the relevant colleges and credentialing bodies,” reflecting similar challenges and pathways as those faced in Canada.

FPESS play an important role in meeting the surgical needs of rural communities in Western and Northern Canada. This is the first evaluation of the Prince Albert ESS program, the only formal 12-month graduate accredited program for ESS in Canada from 2007 to 2017. The goals of this study were to evaluate the ESS training program from the perspective of graduates and to explore the post-training experiences of graduates, including the facilitators and barriers to practicing and maintaining ESS.

## Methods

We administered a structured survey with some open-ended questions (Appendix A) by telephone to graduates of the Prince Albert ESS program 2007 – 2017. We sent an e-mail message to all potential participants from a third party (Rural Physicians Action Program administrator) who also scheduled the survey interviews and completed transcription. Survey interviews took place May – June, 2017 and each one lasted between 45 minutes and 1.5 hours. The study team in collaboration with ESS program staff designed the survey, which consisted of both open and closed ended questions. Survey questions were related to research objectives and the evaluation needs of the ESS program. Survey sections were Demographics, Procedural Training and Current Scope, Privileging, CPD, Mentorship and Participant Assessment of the ESS training program.

We analyzed the qualitative data from the interviews for recurring themes using basic principles of thematic analysis: immersion in the transcripts, development of a codebook, coding of transcripts, and identification of themes.^19^ The lead investigator and a research coordinator read all transcripts and jointly developed a codebook. The research coordinator applied this codebook to the transcripts using NVivo (QSR International) software and then grouped codes under broader themes; these themes ended up matching a priori survey sections. We summarized themes and sub-themes. We selected for inclusion in the report quotations most representative of a thematic response.

The sample was homogenous and almost everyone who went through the ESS training program at Prince Albert participated in this study. Thus, theoretical saturation was reached. The study was approved by the Behavioural Research Ethics Board, University of British Columbia.

## Results

**Figure 1:**
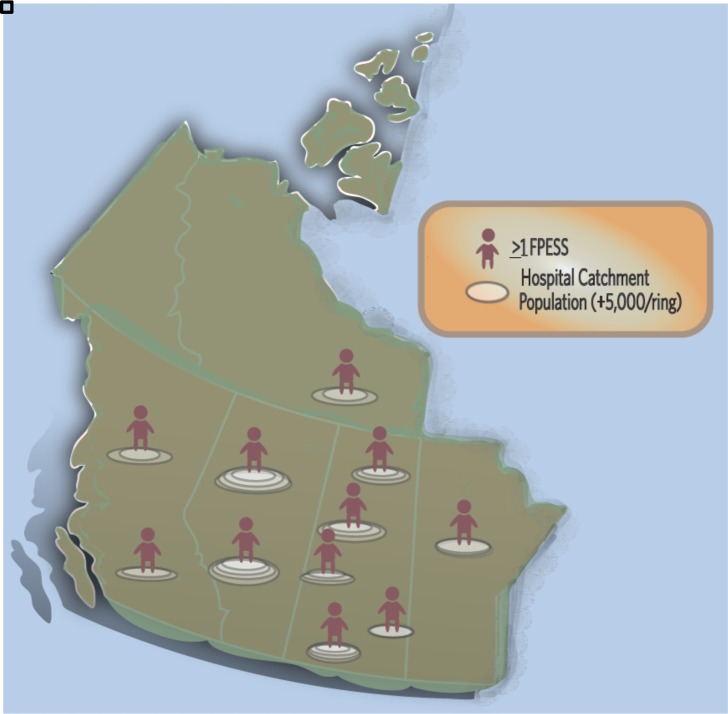
Where FPESS have been practicing after completing the ESS training program

Eleven FPESS graduates participated in the study out of 13. They were all in rural practice in British Columbia (BC), Alberta, Saskatchewan, Manitoba, or the Northwest Territories (Figure 1). Six participants entered the program from rural practice while five entered from residency. The length of time in ESS practice ranged from six months to 8.5 years. The population sizes of the hospital catchments ranged from 7,200 to 20,000. Distance to the nearest surgical centre was between 50 kms and 150 kms for 10/11 respondents; one respondent reported a distance of 1500 km. Only one participant had a CT scanner on-site.

One study participant worked as a solo FPESS in the community; six had one other ESS colleague, three had three other colleagues. No graduate practiced alongside local specialists. However, four were supported through Obstetrical outreach from a regional centre and three were supported by General Surgery outreach. Other outreach included Ear, Nose, Throat (ENT), and Orthopedics. The respondent who worked in the solo model noted that another ESS practitioner was highly desired and two others noted additional ESS support in the community would be of benefit.

Survey results, including thematic summaries of participant comments, are presented below by survey topic. Table 1 lists themes from interviews with the graduates.

**Table 1:** Themes from interviews with graduates of the ESS program

Theme
Procedural training and current scope
Privileging
Continuing Professional Development
Mentorship
Participant assessment

### Procedural Training and Current Scope

Almost all of the graduates interviewed noted that the scope and volume of their training was adequate preparation for independent practice. As one participant noted, “I knew exactly what I needed and got the training I needed.”

Participants reported their surgical volumes within the past year (after completing the ESS training program). The most common procedures performed in community practice were upper GI endoscopy (annual mean: 147, annual range 60-550), colonoscopy (annual mean: 199; annual range 45-550), and caesarean section (annual mean 40: annual range 2 – 300). For the remainder of the major procedures (appendectomy, inguinal hernia, surgical sterilization), the volumes were low. The average annual volume of appendectomies and hernias were 3 and 8 respectively with a range of 1-5 for appendectomies and 3-17 for hernia repair.

There was a close match between the training curriculum and post-training practice (Table 2). Discrepancies were attributed either to lack of population need or to the provision by outreach specialists from regional centres. Other participants found that their practice settings did not have the equipment needed for a procedure.

**Table 2:** Comparison between procedures in ESS training curricula and procedures performed by graduates after completion of the ESS training program (n = number of graduates)

Procedure	Trained	Used in practice
Upper GI endoscopy	11	11
Colonoscopy	11	11
Cesarean Section	11	10
Appendectomy - open	9	8
Laparoscopic surgical sterilization	10	7
Repair 4^th^ degree Perineal Tear	10	6
Inguinal Hernia	9	6
Carpal Tunnel Release	8	5
Appendectomy - laparoscopic	9	4
Tonsillectomy	4	3
Surgical management of ectopic Pregnancy	6	1

Most participants suggested that although there may be other procedures useful in a rural setting (“surgical hand issues,” “anything to do with trauma”), the program was already broad enough and it would be difficult to adequately train for more within the 12-month period.

When asked about local OR availability, five of the respondents reported having one operating room in their local hospital and six noted they had two. Five participants reported their ORs running two days a week (three facilities ran two full days and two ran four half days), one three days/week and one five days/week.

Most participants felt that the volume in their surgical program was sufficient to maintain skills, although as one participant suggested, this volume was “just barely [sufficient].” Another noted, “I think we’d always want more volume and more cases.” One participant who did not think there was enough volume in her/his community to maintain surgical competence noted there would likely be “pushback” from colleagues if they left the community for a locum to increase surgical exposure. Likewise, although the majority of respondents felt their surgical program was sustainable, two respondents expressed concern regarding adequate volume for anesthesia and nursing to remain current.

### Privileging

All physicians with ESS providing procedural care must be privileged (granted permission to perform certain clinical activities) by their local hospital. The majority of participants entered the ESS program with a designated community in mind for rural practice and most noted that they had an understanding with the health authority on anticipated privileges and scope of practice post-training. Concomitantly, the majority of participants did not have difficulties with the privileging process. Challenges that did arise in the privileging process were due primarily to the novelty of ESS locally and the lack of any interprofessional understanding of the letter from the U of S that attests to completion of training (“What does this letter actually mean?”). In some jurisdictions, this was compounded by on-going changes in provincial privileging processes or other administrative guidelines. One participant described requiring a letter from regional department heads attesting to competency in addition to the letter of competency issued from the University of Saskatchewan, which created delays to practice.

For the majority of participants who were able to receive privileges, familiarity with FPESS by others was seen as a significant facilitator This familiarity was due to either practicing in the health authority where training was received or practicing in a community familiar with ESS practice. As one participant said, “I’ve been very lucky. Because [our community] has had a history of doing this….” For others, strong community need for local surgical services was also a facilitator to privileging.

### Continuing Professional Development

One of the key enablers of effective post-training CPD was supportive relationships with mentors through contact “on a daily basis” to affirm learning and acquire new skills. Participants in BC noted the efficacy of a newly instituted Clinical Coaching program which links rural FPESS with regional specialists in a formalized coaching relationship with both virtual linkages and face-to-face practice experience in the rural and regional sites. Only two of the participants in the study reported having experience in regional or tertiary ORs.

Seven participants noted they had incorporated new procedural skills since graduating. Skills acquired included vasectomies, laparoscopic appendectomy, carpal tunnel release, endoscopic polypectomy, induction of labour with oral misoprostol, and minor trauma. For those participants who had incorporated new techniques or procedures since graduating from the PA program, common mechanisms of acquisition included overseas work, Alberta Society for Endoscopic Practice CPD conference, peer-to-peer mentoring and the bi-annual Enhanced Surgical Skills conference in Banff. The only barrier to CPD reported was the inability to leave the community due to clinical (on-call) responsibilities.

When prompted, almost all participants felt that a remote presence platform, in which technology (e.g., cameras) is available to connect teams separated by distance including for training and consultation, would be useful in linking their rural OR with regional specialists. As one participant noted, “I would really like it. I would really like the surgeon to be in the operating room with me if I needed it.”

Recommendations for ESS CPD included system-level suggestions such as the use of remote technology, increased theory in practice (as one participant said, “When *not* to do the procedure”) and opportunities for high volume practice. Others noted the value of specific venues such as the Alberta Society for Endoscopic Practice yearly conference, the Comprehensive Approach to Rural Emergencies course and “Really Rural Surgery” podcasts. Almost half of the respondents noted the value of overseas high-volume experience; several who did not have this opportunity appreciated its potential value but found it infeasible, mainly due to family reasons. One participant noted the lack of facilitation of CPD by her/his health authority to address concerns with competencies due to low volume; this lead to increased stress and negatively affected this her/his overall quality of life. As the participant noted:

[I had a concern with numbers and competency and] …asked for assist time in the tertiary centre partly to get to know my local specialists but also just to keep my skills from totally atrophying. This was not facilitated. So, this meant that at times, I went for 2-3 months without any emergency or booked c-sections. It is hard to keep newly acquired skills up when the volume is so low. But then you’re expected to do a very challenging second stage section in the middle of the night with a resident assisting and no surgical back up in town if anything goes wrong. I spent the first few months paralyzed by fear and completely stressed about work which impacted my health, sleep, relationships…

### Mentorship

The mentorship received from faculty in the Prince Albert Program was highly regarded by all participants, as was the role of the support staff (although one participant mentioned that they were under-resourced). There was more variation in perception regarding the supportiveness of the U of S Department of Surgery with three participants responding they were not supportive due to lack of contact.

Outside of the Clinical Coaching program in BC, graduates of the PA program were not successful in establishing mentoring relationships with their regional referral centres. Participants reported that barriers to establishing supportive mentoring relationships outside of the program were rooted in distrust and misunderstanding. As one participant noted, “Lack of understanding for ESS breeds a sense of threat or mistrust.”

### Participant assessment

Participant feedback about their experiences in the PA program was overwhelmingly positive with *flexibility* and, as noted above, *support by faculty* being the most important attributes. Flexibility was seen as that which allowed learning to be tailored to the needs of the individual learner and community to which they would return (a heightened sense of flexibility was noted by those who went through the program earlier). This flexibility was linked by some to a sense of openness: “The General Surgeons didn’t understand my role at the end, but they were welcoming and motivated to teach even though they didn’t understand fully.” Faculty support was also manifest through the responsibility the trainees felt they were given and “the enthusiasm of the Obstetricians and General Surgeons for training us.” In a representative summary, one participant said:

*Cannot say anything bad about [the program] – cherished it. The immersion in it was really great, the fact there’s only two ESS residents in the whole hospital, everyone quickly figures out who you are. Therefore, the opportunities to get involved are more. The volume was good. Appropriate patient selection*.

Additional comments included “I had such a good experience that year, and even exceeded my expectations” and “Best department that I worked in. They just wanted me to learn new things all the time. The endoscopy training was fantastic; the volume amount was the great way to make sure I succeeded.”

Suggestions for improvement focused on increased volume of procedures and expanded educational modalities. Several participants specifically noted the desire for increased volume for endoscopy training and others noted the value of moving to a competency-based program: (“I would like to see it more of a competency-based program. And stay in the program until you are comfortable (and not a minimum pass, etc.) and have the confidence.”). Another participant noted the need for prioritization of ESS learners for OR time: “I understand [specialist residents] need experience and tissue time, but they have five years to acquire this and we have one year.”

Participants suggested several ways to augment the core curriculum including a structured reading program, seminars with practicing ESS physicians to understand to a larger practice context, surgical office time (“I had to ask a lot of questions about pre and post-operative stuff when I got back, because I was only doing the surgery”), training in rural communities by shadowing physicians with variation in scope of practice, simulation training outside of the OR and a set plan for post-training CME. A further suggestion was increased program organization (“It’s a little bit chaotic”). The final more substantive suggestion regarded the perceived need for a stronger political voice for the Prince Albert Surgical Department in academic and provincial discussions around workforce training.

When asked for their assessment of the future of enhanced surgical skills in Canada, participant responses included concern over costs in a climate of fiscal constraint, the impact of the low numbers of FPESS working on call and consequently on sustainability, and difficulties recruiting and maintaining the limited number of proceduralists that currently practice. Several participants believed the future of ESS will depend on the quality of their reputation: “It will be a struggle, but as long as the ESS student keeps performing high quality work, they will gradually build their reputation.”

## Discussion

The total potential participant cohort for this study was 13 and we reached 11, close to the population, allowing us to capture enough variation in response to represent experiences of the program adequately. The consistency of the responses across participants allows some tentative conclusions to be drawn, namely that overall, the Prince Albert ESS training program, through the lens of its graduates, was successful in meeting the training needs of learners and preparing them for rural practice. Beyond experiences with the program, however, further reflections of participants regarding 1) post-training privileging for practice, 2) on-going relationships with specialists, and 3) the lack of formalized continuing medical education suggest larger systemic issues, centered in the post training reality of the ESS graduates.

Graduates of the ESS program do not receive official accreditation or designation after completing the program. They receive a letter of completion from the program as evidence of the demonstration of individual competence in a high-quality training program. The comparison would be the Category 1 programs in Emergency Medicine and Family Practice Anesthesia, each with their own Certificate of Added Competence (CAC) from the College of Family Physicians of Canada (CFPC). The present collaborative process between the Royal College of Physicians and Surgeons of Canada (RCPSC) and the CFPC to elevate ESS programs to Category 1 status promises to unlock the postgraduate privileging process and build support for ESS among specialist colleagues.^20^ This would also help to address the significant challenge of privileging of FPESS, not only inter-provincially but also intra-provincially, that is, a physician who has permission to perform a set of clinical activities in one health authority may not be able to obtain this same permission if they move to another health authority.

If the recommendations for networked rural surgical care from the Joint Position Paper are to be realized, the historical frequently dysfunctional relationships between specialist and FPESS will need to grow into the relationships of trust and collaboration described as the foundation for successful network.^21^ The new Clinical Coaching programs, proposed in the Rural Surgery and Obstetric Networks in BC, offer an evidence based model within which to grow these relationships.

The small cohort of ESS participants, the heterogeneity of its practice scope, the barriers to leaving the community even for a short time, the current lack of support and understanding within the specialist surgical societies, together pose large challenges to CPD for ESS. While volumes in training were considered to be sufficient for competency, the low volume nature of a rural generalist surgical practice was clearly a threat to sustaining currency amongst the graduates. We imagine that Clinical Coaching programs, remote presence, and CQI programs will mitigate the challenges posed by low volume. The global surgical experiences sought by many FPESS graduates offer CPD experiences that pose disruptive challenges to traditional CPD programming and introduce new ethical challenges.

Outside of endoscopy, colonoscopy, and caesarean section, the procedural volumes were low. These findings are a step towards updating existing literature on surgical procedural volumes in rural Canadian jurisdictions.^22,23^ The sustainability of rural FPESS will require, in addition to the improved CPD needs already highlighted, robust Continuous Quality Improvement programs. In a low volume generalist environment, measuring quality might require the documentation and examination of outcomes for 100% of all surgical procedures. It is promising that the new Rural Surgical and Obstetrical Networks program in BC proposes to do just that – reporting and examining all outcomes using a thoughtful reflective privatized self corrective methodology.

### Conclusion

There is broad satisfaction among the graduates of the PA program with the scope and quality of the 12-month program. They developed, and continue to hold, strong mentoring relationships with the faculty. The concordance between the curriculum and their scope of practice attests to the suitability of the program to future practice. Their post training challenges with obtaining permission to conduct procedures and with forming collaborative relationships with specialist surgeons might be resolved, at least partly, with the present collaborative efforts between the two licensing Colleges to elevate ESS to Category 1 and to award a Certificate of Added Competency to its graduates. Concomitantly, this may lead to greater awareness and understanding of ESS practice by the specialties and foster stronger and more collaborative relationships between providers. This leaves the urgent need for effective rurally appropriate post-training CPD opportunities as the outstanding challenge to the sustainability of FPESS.

## Appendix A Survey

### Prince Albert ESS program Graduates 2007-2015 Oral Survey

#### Personal Information and Background

1. Gender______ Year of Birth_______ Length of time in ESS practice __________
2. Year you graduated from PA program ___________

2. Present Practice: Rural___ Includes ESS____
3. Present Practice: Province___ Hospital Catchment pop____ Distance to a larger surg program ____
4. Number of local surgical practices:
  ESS (other than yourself)_____
  Gen Sx: local_____Outreach______
  OBGYN: local____Outreach___
  Other: local____ outreach______
5. How many OR theatres in your hospital____
  for a typical week how many theatres are running____
  for how many days/.5 day____
  Does your hospital have a CT scanner_____
6. Have you practiced ESS in other communities after training___ which___ (same questions about demographics for prev communities)
7. Entered ESS Program : directly from Residency_________from rural practice________other____

#### Privileging

8. Entered ESS Program with a designated rural community for future ESS practice: Yes____ No_____
  If Yes
  8.A) Did you have an understanding with the HA_____ and local medical staff____ on anticipated privileges/scope of practice after training?
  8.B) What difficulties, if any, did you encounter with the HA____ Medical Staff____ when you returned.
  8.C) Have these been resolved? How?
10. Did the local Medical Director accept your PA letter as evidence of competency, or did s/he insist that your training be verified by local specialist staff?
11. For those without privileging issues, what facilitated ? Local culture of ESS already there? Other?

#### Enhanced Surgical Skills

15. (a) Which procedures did you train for? (b) What procedures are you currently using? (c) What is your annual volume?
  1. Upper GI endoscopy
  2. Colonoscopy
  3. Appendectomy
    Open
    Laparoscopic
  4. Inguinal Hernia
  5. Laparoscopic surgical sterilization
  6. Carpal Tunnel Release
  7. Tonsillectomy
  8. Cesarean Section
  9. Repair 4^th^ degree Perineal Tear
  10. Surgical mgmt. Ectopic Pregnancy
    Open
    Laparoscopic
  11. Vascectomy
  12. Hydrocealectomy
  13. Endometrial ablation
  14. Eschers
  15. Umbilical hernia repairs
  16. Neonatal circumsicions
  17. Post partum tubal ligations
  18. D&C’s (miscarriage/incomplete abortion)
16. In your opinion, is the volume of surgery in your program sufficient to sustain
  1. surgical skills
  2. anesthesia skills
  3. OR nursing skills?
17. For those skills in which you received training, are there any for which you felt the training was an inadequate preparation for independent practice?
18. For those skills in which you were trained but do not include in your practice, is this due to:
  - The procedure being provided locally by another ESS____
  - a specialist____(local or outreach)
  - the procedure not being supported locally by the hospital______(because of cost/equipment___privileging issues___)
19. Are there procedures which you feel, in retrospect, need not/should not be included in the training?
20. Are there procedures for which you wish you had received a larger volume of training?
21. Are there procedures which are not included in the PA program but which you believe should be considered for inclusion (eg trauma surgery)?

#### CPD

22. Have you incorporated any new techniques or procedures into your practice since graduating?
23. How did you adquire these skills?
24. Have you attended the Banff CME for ESS in
  - 2007
  - 2009
  - 2012
  - 2014
  - 2016
25. Have you attended other CME relevant to ESS? Please describe?
26. Have you had any opportunity to experience CPD in a regional or teretiary OR?
27. If yes, please describe the details and how you obtained this.
28. If no, please tell us if you sought this opportunity at ay time?
29. Do you have recommendations for CPD for ESS?
30. Is a high volume overseas experience something you:
  - did after training
  - considered, but didn’t do
  - had other interests
31. If you didn’t do it, was it due to
  - financial reasons
  - family reasons
  - other interests

#### Mentorship

32. Have you been successful in establishing a supportive mentoring relationship with:
  - A General Surgeon_____(present community_____other communities___)
  - An OBGYN______
  - Other specialist surgeons_____
33. If yes, what do you see essential to the relationship? (What makes it work?)
32. If no, what do you see as the barriers to these relationships?

#### Improving the program

33. What worked well?
34. What could be improved?
35. What needs to go?
36. Were the faculty helpful/supportive?
37. Were the support staff/helpful/support?
38. Was the Department of Sur/MEDICINE helpful/supportive?
39. Did you have a good relationship with faculty?

#### Interprofessional Relationships

40. What is your perception of current attitudes towards ESS (from specialists, generalist colleagues, administrators)? Do you think attitudes have changes since your received training?
41. Do you have any additional comments?
